# Clinical features and genetic spectrum of Chinese patients with hereditary spastic paraplegia: A 14-year study

**DOI:** 10.3389/fgene.2023.1085442

**Published:** 2023-02-27

**Authors:** Weiyi Yu, Ji He, Xiangyi Liu, Jieying Wu, Xiying Cai, Yingshuang Zhang, Xiaoxuan Liu, Dongsheng Fan

**Affiliations:** ^1^ Department of Neurology, Peking University Third Hospital, Beijing, China; ^2^ Beijing Municipal Key Laboratory of Biomarker and Translational Research in Neurodegenerative Diseases, Beijing, China; ^3^ Key Laboratory for Neuroscience, National Health Commission, Ministry of Education, Peking University, Beijing, China; ^4^ School of Basic Medical Sciences, Peking University, Beijing, China

**Keywords:** hereditary spastic paraplegia, China, genetic spectrum, follow-up, heterogeneity

## Abstract

**Background:** Hereditary spastic paraplegia (HSP) constitutes a group of clinically and genetically rare neurodegenerative diseases characterized by progressive corticospinal tract degeneration. The phenotypes and genotypes of HSP are still expanding. In this study, we aimed to analyse the differential diagnosis, clinical features, and genetic distributions of a Chinese HSP patients in a 14-year cohort and to improve our understanding of the disease.

**Methods:** The clinical data of patients with a primary diagnosis of HSP at the initial visit to the Department of the Neurology, Peking University Third Hospital, from 2008 to 2022 were retrospectively collected. Next-generation sequencing gene panels (NGS) combined with a multiplex ligation-amplification assay (MLPA) were conducted. Epidemiological and clinical features and candidate variants in HSP-related genes were analyzed and summarized.

**Results:** 54 cases (probands from 25 different pedigrees and 29 sporadic cases) from 95 patients with a primary diagnosis of HSP were finally confirmed to have a clinical diagnosis of HSP based on clinical criteria, including their clinical findings, family history and long-term follow-up. Earlier disease onset was associated with longer diagnostic delay and longer disease duration and was associated with a lower risk of loss of ability to walk independently. In addition, 20 candidate variants in reported HSP-related genes were identified in these clinically diagnosed HSP patients, including variants in *SPAST, ALT1, WASHC5, SPG11, B4GALNT1,* and *REEP1*. The genetic diagnostic rate in these 54 patients was 35.18%.

**Conclusion:** Hereditary spastic paraplegia has high clinical and genetic heterogeneity and is prone to misdiagnosis. Long-term follow-up and genetic testing can partially assist in diagnosing HSP. Our study summarized the clinical features of Chinese HSP patients in a 14-year cohort, expanded the genotype spectrum, and improved our understanding of the disease.

## Introduction

Hereditary spastic paraplegia (HSP) is a group of neurodegenerative diseases characterized by progressive degeneration of the corticospinal tract. The prevalence of the disease is 0.1–9.6 per 100,000 people (Meyyazhagan and Orlacchio, 2022). The clinical manifestation starts in infancy and continues into adulthood with slow progression (Fink, 2013; Shi et al., 2022). HSP can be divided into pure and complex (or complicated) phenotypes based on clinical symptoms (Harding, 1983). Patients with pure hereditary spastic paraplegia (p-HSP) mainly present with lower limb spasticity and weakness; speech and patient survival are unaffected. Complicated hereditary spastic paraplegia or complex hereditary spastic paraplegia (c-HSP) can also be associated with extrapyramidal symptoms, cerebellar or cognitive dysfunction and peripheral neuropathy. The survival of c-HSP patients is often related to the severity of other non-limb symptoms (Coutinho et al., 2013; Fink, 2013; Dong et al., 2018). The disease has been associated with genetic factors, and familial cases are more common than sporadic cases, with a ratio of approximately 2:1 (Di Fabio et al., 2016). With the progression of molecular diagnostic techniques, more than 80 causative mutations have been identified, and the number is increasing (Meyyazhagan and Orlacchio, 2022). HSP can be classified into SPG1–SPG83 based on the spastic paraplegia gene (SPG) locus and the discovery order. Common modes of inheritance include autosomal dominant (AD), autosomal recessive (AR), and X-linked dominant. SPG4, SPG3A, and SPG31 are the most common causes of AD-HSP, while SPG5, SPG7, and SPG11 are common in autosomal recessive spastic paraplegia (AR-HSP) (Méreaux et al., 2022). However, the distribution frequency of gene mutations varies in different regions in the world (Saputra and Kumar, 2021). For example, SPG 8 and SPG31 are rare in China (Xing and Du, 2022), and SPG42 has been found in Chinese families but not in large cohorts in Europe (Schlipf et al., 2010).

HSP has high clinical and genetic heterogeneity, overlapping with other neurodegenerative diseases, such as amyotrophic lateral sclerosis (ALS), primary lateral sclerosis (PLS), and Parkinson’s disease (PD) (Elert-Dobkowska et al., 2019; Saputra and Kumar, 2021). Therefore, the diagnosis of HSP is difficult and partly relies on genetic testing and exclusion diagnostics. Moreover, some clinical observations from smaller-scale studies are insufficiently substantiated by statistical evidence, and few of them had a long-term follow-up. This study aimed to provide a new perspective for the diagnosis and genetic distribution of HSP in China and expand the genotypic spectrum based on a relatively large and long-term cohort.

## Materials and methods

### Patients with a primary diagnosis of HSP: Primary screening

We first searched cases with a primary diagnosis of probable or possible HSP at the initial visit from 2008 to 2022 in our long-term cohort of neurogenerative diseases approved by the ethics committees of Peking University Third Hospital (number: 2008009). These patients met the following criteria [according to Harding criteria (Harding, 1983)] at the initial visit.

### Inclusion criteria

1 Clinical manifestations: The clinical manifestations are spastic paraplegia of both lower limbs, but cases with increased tendon reflexes, slight impairment of rapid alternating movements or mild distal amyotrophy in the upper limbs are not excluded; the cranial nerves and language are not affected.
2 Neurological physical examination revealed spastic gait and positive pyramidal signs.
3 Laboratory testing: Common biomarkers are normal.
4 Imaging: Brain and spinal cord magnetic resonance imaging (MRI) are usually normal, but some patients may present with dysgenesis of the corpus callosum or tapering of the spinal cord.
5 Family history: Usually, positive but not essential.
6 Informed consent was obtained.

#### Exclusion criteria

1 Other common diseases cause spastic paraplegia, such as cerebrovascular diseases, structural damage to the spinal cord, and autoimmune diseases.
2 Disability caused by severe trauma or major surgery.
3 Decline to participate.

### Clinical data collection

Medical records were collected to extract clinically relevant data, including the results of auxiliary examinations, such as biochemical tests, neurophysiological examination and MRI. To retrospectively quantify the clinical severity of motor syndromes, we used the Four-Stage Functional Mobility Score (1 = mild symptoms walking without an aid; 2 = walking without aid but unable to run; 3 = walking with aid; and 4 = wheelchair-dependent) (Ebrahimi-Fakhari et al., 2020). The follow-up data of the patients *via* outpatient visits, inpatient stays and telephone interviews were collected by clinicians in the Department of Neurology, Peking University Third Hospital. Patients who could not be successfully followed up for three different time periods were defined as lost to follow-up. The final follow-up deadline for this study was August 2022. Written informed consent was obtained from all study participants.

### Patients with a final diagnosis of HSP: Secondary screening

**FIGURE 1:**
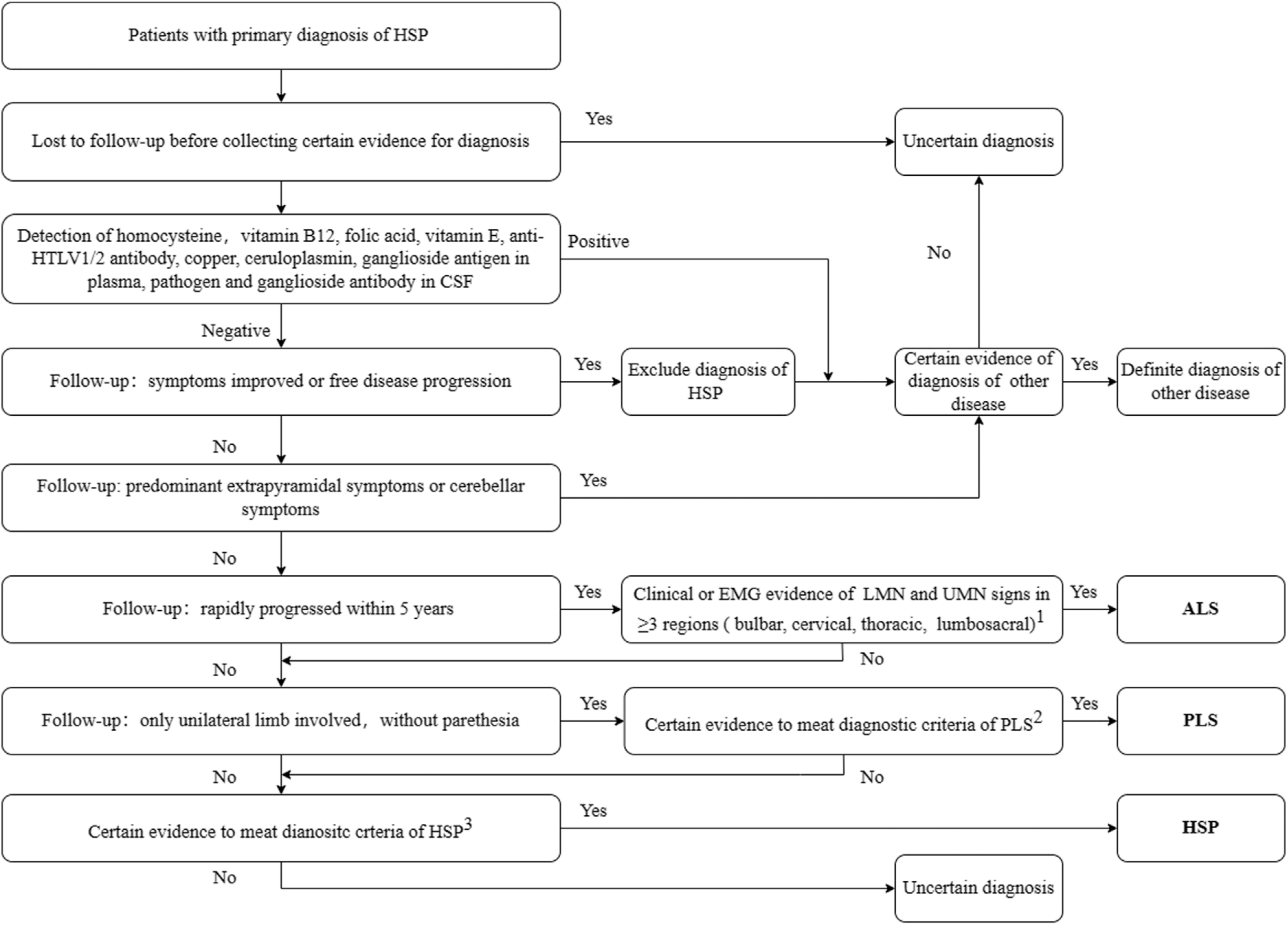
Diagnostic workflow for secondary screening. 1. Awaji-shima Criteria (Kiernan et al., 2011); 2. Consensus diagnostic criteria for PLS (Turner et al., 2020); 3. Criteria for HSP (Harding, 1983). HSP, hereditary spastic paraplegia; ALS, amyotrophic lateral sclerosis; PLS, primary lateral sclerosis; HTLV, Human T-lymphotropic virus; LMN, lower motor neuron; UMN, upper motor neuron; EMG, electromyogram; CSF, cerebro-spinal fluid. Other diseases include Parkinson’s disease, spinocerebellar ataxia, copper deficiency myelopathy, peripheral neuropathy, and chronic neurobrucellosis.

At least two senior neurologists reviewed the clinical data including the follow-up data, and confirmed the final diagnosis using the clinical guidelines for the corresponding diseases. Patients fulfilling the clinical diagnostic criteria for HSP (Harding criteria (Harding, 1983)) and our diagnosis procedures were diagnosed with HSP, irrespective of their genetic diagnosis. The diagnostic workflow is shown in Figure 1. Patients with a final diagnosis of other diseases or with an uncertain diagnosis due to lack of sufficient evidence were excluded from the following study.

### Gene sequencing

Genetic testing was performed to support the clinical diagnosis and for investigations. After obtaining the consent of the patient or his or her legal guardian, peripheral blood was collected, and DNA was extracted. Genetic testing was performed using next-generation sequencing (NGS) combined with a multiplex ligation-dependent probe amplification assay (MLPA). A commercial panel (Polyneuropathy and Spastic Paraplegia Panel, LM-NE0803)from Beijing Kangso Medical Laboratory including genes related to HSP was applied in 57 paitents. Detected genes are listed in the supplemental materials. Whole exome sequencing (detected in Beijing Kangso Medical Laboratory and RunningGene Inc.) was applied in 25 patients. MLPA was also applied. 13 patients refused the genetic testing. All variants were assigned by the 1000 Genomes Project (http://phase3browser.1000genomes.org/index.html), exome sequencing projects (http://evs.gs.washington.edu/EVS/), and the Exome Aggregation Consortium database (Fig. http://exac.broadinstitute.org/) in OneStep filtration. SIFT (http://sift.jcvi.org/), Polyphen2 (http://genetics.bwh.harvard.edu/pph2/), Mutation Taster (http://www.mutationtaster.org/), dbSNP (https://www.ncbi.nlm.nih.gov/projects/SNP) and ClinVar databases (https://www.ncbi.nlm.nih.gov/clinvar/). The evolutionary conservation score of the mutation and its function were predicted, and the pathogenicity of the mutation was assessed based on the ACMG guidelines. The results and the available family samples were validated by Sanger sequencing. Finally, the causative mutations were compared with the human gene mutation database (http://www.hgmd.cf.ac.uk/) to determine whether they had been reported.

### Statistical analysis

SPSS 19.0 was used for statistics and analysis of the data. Demographic and clinical variables were characterized by descriptive statistics, expressed as the mean and standard deviation (SD) for continuous variables and as frequency counts and percentages (with 2 significant figures) for categorical variables. Continuous variables were analyzed using an independent *t*-test or Mann–Whitney *U* test, and groups were compared by Fisher’s test or chi-square test for categorical variables. Analysis of variance (ANOVA) and LSD *post hoc* tests were used to compare the three groups. Kaplan-Meier analysis were performed to assess the risk of walking disability, and Log-rank test was performed to analyze the differences among the subgroups. *p* < 0.05 was considered statistically significant.

## Results

### Distribution of final diagnosis and clinical features of HSP patients

**FIGURE 2:**
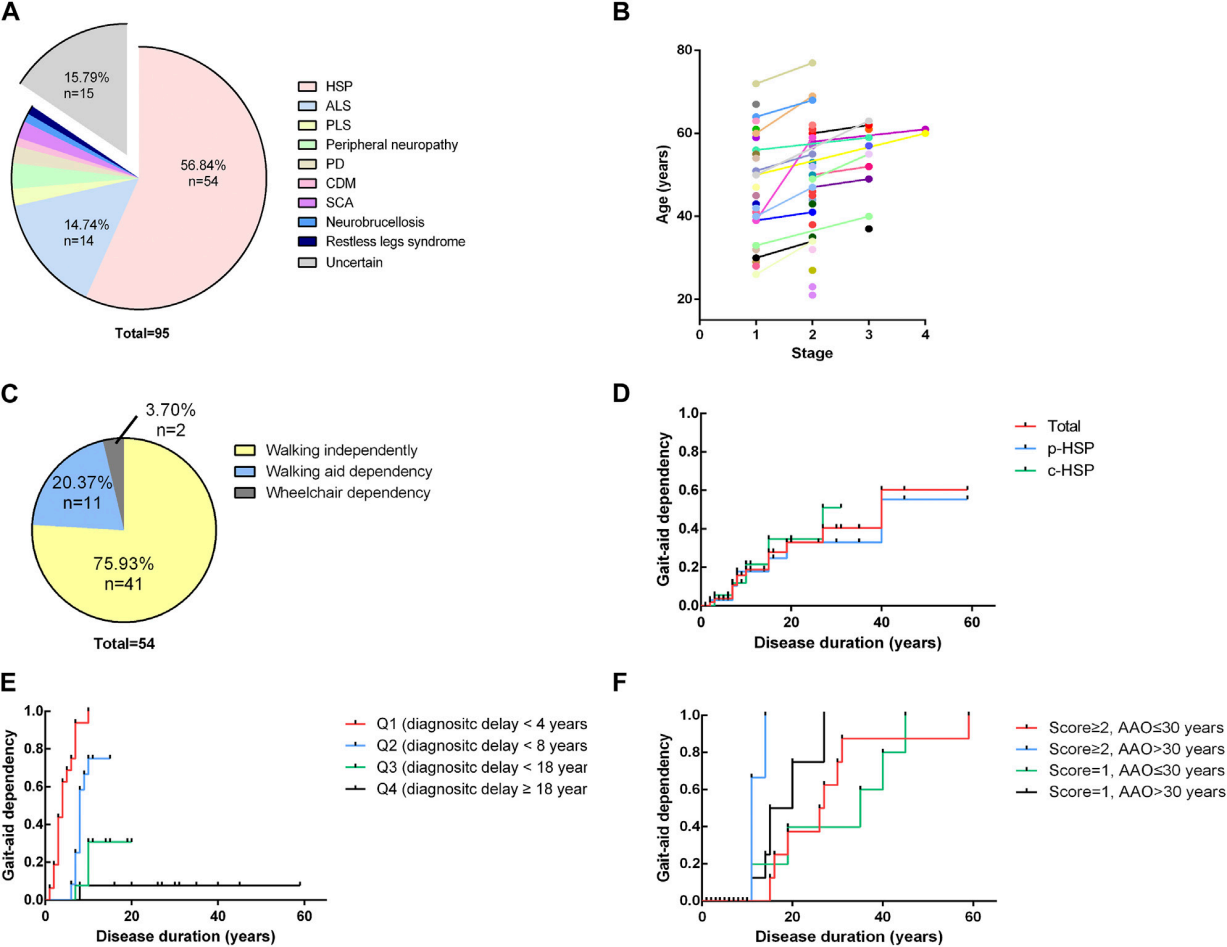
Distribution of final diagnosis, disease severity and Kaplan-Meier analysis **(A)**. Distribution of final diagnoses. A total of 95 cases (only probands and sporadic cases were included) with a primary diagnosis of HSP were found, and HSP was ultimately diagnosed in 54 patients. Other final diagnoses included amyotrophic lateral sclerosis (ALS) (*n* = 14), peripheral neuropathy (n = 3), primary lateral sclerosis (*n* = 2), Parkinsonism disease (*n* = 2), spinocerebellar ataxia (SCA) (*n* = 2), copper deficiency myelopathy (CDM) (*n* = 1), restless legs syndrome (*n* = 1) and chronic neurobrucellosis (*n* = 1). ALS, amyotrophic lateral sclerosis; PLS, primary lateral sclerosis; PD, Parkinson’s disease; CDM, copper deficiency myelopathy; SCA, spinocerebellar ataxia. **(B)**. Four-stage functional mobility score assessed at the age at the initial visit and the time of final follow-up. Patients are shown with different colors with lines connecting different time points. A general trend toward higher scores with increasing age becomes apparent. **(C)** Distribution of the ability to walk of the patients at the time of the evaluation for the study. 75.93% of patients (*n* = 41) were still able to walk for at least 10 m without aid, and 20.37% of patients (*n* = 11) required an aid to walk. Only 2 patients (2/54, 3.70%) had completely lost their ability to walk and had to use wheelchairs in daily life. **(D)** Kaplan-Meier analysis of loss of independent walking based on clinical phenotypes. The time course of walking aid dependency of each clinical subgroup is virtually indistinguishable from that of the total cohort. **(E)** Influence of diagnostic delay on independent walking. The total cohort was divided into 4 subgroups of equal size according to their years of diagnostic delay (Q1 < 4 years, Q2 < 8 years, Q3 < 18 years, Q4 ≥18 years). Shorter diagnostic delay (time from onset to initial visit) is associated with a higher risk of becoming walking aid dependent earlier in the disease course (*p* < 0.0001). **(F)** Influence of the age of onset and disease severity at the initial visit. The total cohort was equally divided into four groups according to the combination of the four-stage functional mobility score (score = 1 or ≥2) and age at onset. Later onset and higher baseline functional mobility scores were associated with a higher risk of disability of walking independently earlier in the disease course (*p* = 0.0011).

A total of 95 cases (only probands and sporadic cases were included) with a primary diagnosis of HSP were found, and HSP was ultimately diagnosed in 54 patients. Other final diagnoses included amyotrophic lateral sclerosis, peripheral neuropathy, primary lateral sclerosis, early-onset Parkinsonism disease, spinocerebellar ataxia, copper deficiency myelopathy, restless legs syndrome and chronic neurobrucellosis. 15 patients had no definite diagnosis due to a lack of certain evidence especially those who were lost to follow up. Among them, 1 patient was excluded from HSP because his symptoms improved. The distribution of the final diagnosis is shown in Figure 2A. The demographic and clinical features of these 95 patients and the detailed information of all cases are listed in Supplementary Tables S1, S2 in the supplemental materials. The 54 patients were probands from 25 different pedigrees and 29 sporadic cases. The 54 patients had a mean age of onset of 33.24 years, a mean diagnostic delay of 12.59 years and a mean disease duration of 13.85 years. In terms of inheritance features, 29 patients were sporadic, 2 patients had AR inheritance, and 23 patients had AD inheritance (Table 1). There was no statistically significant difference in the age at onset, years of diagnostic delay (time from age of onset to age at the initial visit), or disease duration between the two sexes.

**TABLE 1:** Demographic, clinical features and progression of patients with clinical HSP compared by clinical phenotype.

	All HSP (*n* = 54)	Pure-HSP (*n* = 35)	Complex-HSP (*n* = 19)	*p*-value (compared by phenotype)
Sex (Male, %)	72.22	65.71%	84.21	n.s
Age at onset, y	33.24 (1–62)	34.00 (1–62)	31.84 (8–59)	n.s
Age at initial visit, y	45.83 (20–72)	47.69 (20–72)	42.42 (21–61)	n.s
Diagnostic delay, y	12.59 (0–56)	13.69 (1–56)	10.58 (0–30)	n.s
Disease duration, y	13.85 (1–59)	14.51 (1–59)	12.47 (2–31)	n.s
Mode of inheritance, n				
Dominant	23	17	6	D vs. R: n.s
Recessive	2	1	1	R vs. S: n.s
Sporadic	29	17	12	D vs. S: n.s
Gait-aid use, n (%)	13 (24.07)	9 (25.71)	4 (21.06)	n.s
Cane/walker	11 (20.37)	8 (22.86)	3 (15.79)	
Wheelchair	2 (3.70)	1 (2.86)	1 (5.26)	
Time to gait-aid use, y	12.92 (2–40)	13.25 (2–40)	12.40 (3–27)	n.s

A total of 64.81% of patients presented with p-HSP (*n* = 35), and 35.19% presented with c-HSP (*n* = 19). The first symptoms of the patients were mainly described as difficulty walking, including shuffling gait caused by rigidity of the lower limbs, decreased walking distance, and weakness in the lower limbs. Among all the HSP patients, 11 patients had blader dysfunctions, including urinary urgency, frequency, incontinence. Peripheral neuropathy was the most common presenting symptom (*n* = 11, 57.90%) in c-HSP patients. Other accompanying symptoms included ataxia (*n* = 6), dysarthria (*n* = 1), and cognitive disturbance (*n* = 1). Clinical features are summarized and analyzed based on clinical phenotypes in Table 1. There was no statistically significant difference in either clinical feature between the two phenotypes in our cohort. Considering that most of the patients admitted to our department were adults, we compared the disease duration and years of diagnostic delay based on the AAO (AAO<18 years *versus* AAO≥18 years). The results in Table 2 show that there was a statistically significant difference in either disease duration or diagnostic delay between the two subgroups.

**TABLE 2:** Distribution and progression of patients with clinical HSP compared by age at onset.

	All HSP (*n* = 54)	Preadult (age<18 years) onset	Adult (age≥18 years) onset	*p*-value (compared by phenotype)
		(*n* = 8)	(*n* = 46)	
Sex (Male, %)	72.2	87.50	69.57	n.s
Diagnostic delay, y	12.59 (0–56)	24.38 (5–56)	10.54 (0–40)	0.001
Disease duration, y	13.85 (1–59)	24.25 (6–59)	11.98 (1–40)	0.005
Gait-aid use, n (%)	13 (24.07)	0	13 (21.06)	—
Cane/walker	11 (20.37)	0	11 (15.79)	
Wheelchair	2 (3.70)	0	2 (5.26)	

Abbreviation: HSP, hereditary spastic paraplegia; n. s., not significant; OR , odds ratio; D, dominant; R, recessive; S, sporadic; vs, *versus*; CI, confidence interval.

### Age of onset and diagnostic delay were associated with loss of independent walking

At the time of the evaluation for the study (mean disease duration = 13.85 years, median disease duration = 10 years), 75.93% of patients (*n* = 41) were still able to walk for at least 10 m without aid, and 20.37% of patients (*n* = 11) required an aid to walk. Their mean age at onset of gait aid use of 52.54 years. Only 2 patients (2/54, 3.70%) had completely lost their ability to walk and had to use wheelchairs in daily life (Figure 2C). After their disease durations of 10/20/30 years, 55.56/66.67/90.74% of patients regularly used a walking aid. There was no statistically significant difference in the outcomes or time to gait aid use between the two clinical phenotypes or modes of inheritance. We used the four-stage functional mobility score to quantify the clinical severity of motor syndromes at the age at the initial visit and the final follow-up. A general trend toward higher scores with increasing age becomes apparent, indicating that symptoms may show a relatively non-progressive course in early-onset patients (Figure 2B).

We also performed a Kaplan-Meier analysis to assess the risk of becoming dependent on an aid for ambulation, including a cane, walker and wheelchair. The time course of walking aid dependency of each clinical subgroup was virtually indistinguishable from that of the total cohort (Figure 2D). The total cohort was divided into 4 subgroups of equal size according to their years of diagnostic delay (Q1 < 4 years, Q2 < 8 years, Q3 < 18 years, Q4≥18 years). Shorter diagnostic delay (time from onset to initial visit) was associated with a higher risk of becoming walking aid dependent earlier in the disease course (*p* < 0.0001) (Figure 2E). As we mentioned above, younger patients were found to have a longer diagnostic delay and all of the patients with an AAO<18 years walked independently of aid in our cohort (Table 2). Moreover, patients particularly delayed presentation when they were subjectively asymptomatic or had mild symptoms. These may be attributed to the diagnostic delay and affect baseline functional mobility. Therefore, we divided the total cohort into 4 subgroups according to the combination of the four-stage functional mobility score (score = 1 or ≥2) at baseline (initial visit) and age at onset of 30 years (for equal size) (Figure 2F). The results showed that later onset and higher baseline functional mobility scores were associated with a higher risk of disability of walking independently earlier in the disease course (*p* = 0.0011). Patients with early age at onset were able to maintain independent walking for longer periods.

### Genetic analysis and genotype distribution

**FIGURE 3:**
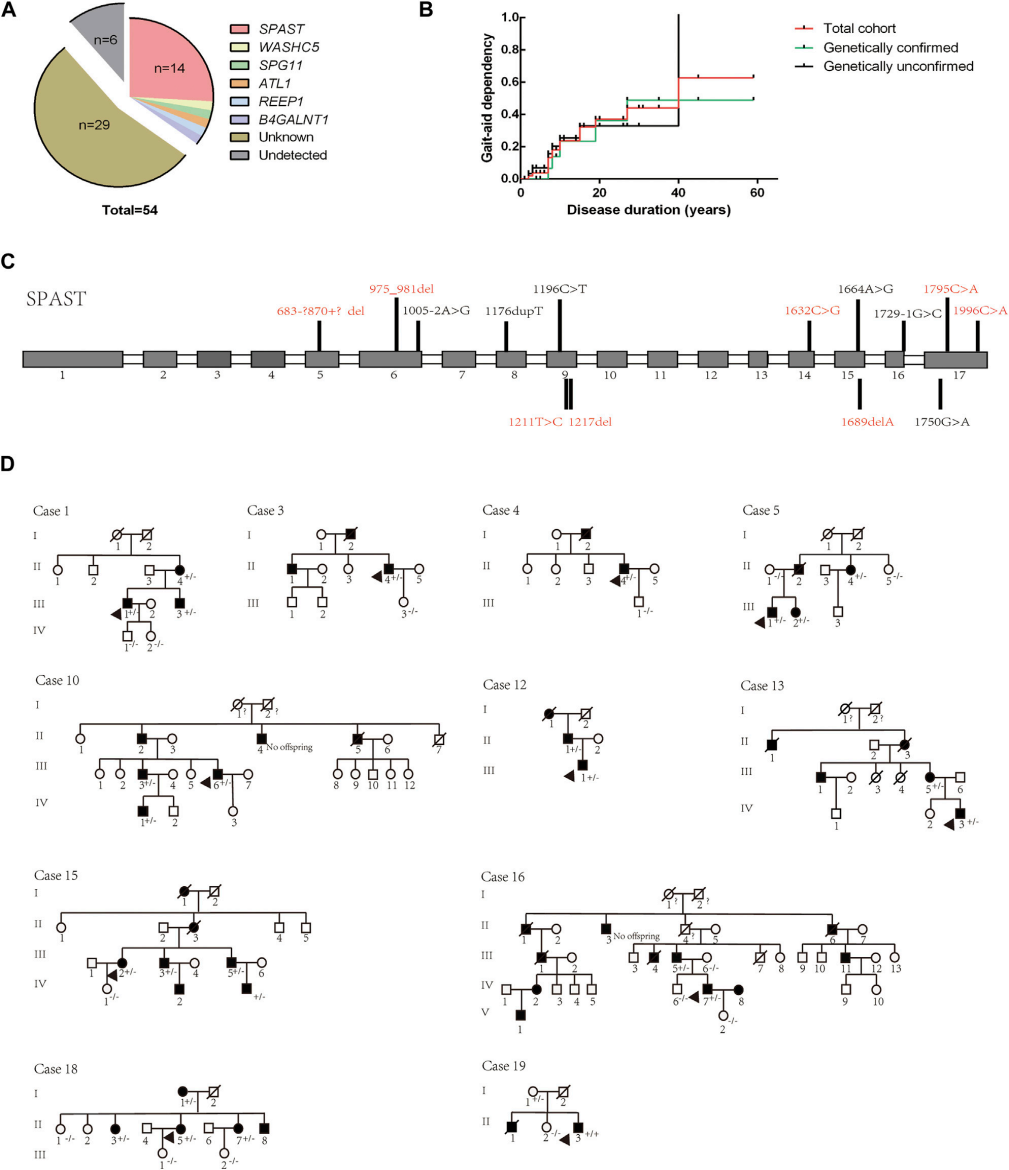
Genetic distribution and pedigree diagrams. **(A)** Genotype distribution. **(B).** Kaplan-Meier analysis. Patients were divided into two subgroups according to whether they were genetically diagnosed. The time course of gait-aid dependency of the two subgroups shows no difference from the total cohort. **(C)** Detected variants of the *SPAST* gene in our cohort. Unreported variants are shown in red. Gray squares represent exons and white squares represent introns. **(D)** Pedigree diagrams. The diagram numbers correspond to Table 2. Arrows indicate the proband in the pedigree. +/- indicates the affected family member carry a heterozygous mutation, −/− indicates that the family member did not carry the mutation.

NGS combined with MLPA detection detected a total of 19 patients (probands of the pedigrees and sporadic cases) carrying pathogenic or likely pathogenic variants in known HSP-related genes. The results were verified by Sanger sequencing. Cosegregation of these mutations with symptoms was observed within the provided pedigree. The genetic distribution is shown in Figure 3A, and pedigree diagrams and validation are shown in Figure 3D. Clinical features and information of the variants are summarized in Table 3. A total of 39.58% of patients with a clinical diagnosis of HSP were genetically diagnosed (19/48, 6 patients refused genetic testing). Kaplan-Meier analysis was performed to compare the disease course of the genetically confirmed cases or the genetically unconfirmed cases with the total cohort, and the time course of gait-aid dependency of each subgroup was virtually indistinguishable from the total cohort (Figure 3B).

**TABLE 3:** Clinical features of 19 probands or sporadic cases and the variants information.

Case	Sex	AAO (years)	DD (years)	Pheno-type	Additional features	Inheri-tance	Gene	Variant	Het/Hom	ACMG	Refer-ence
1	M	28	11	Pure	—	AD	*SPAST*	c.1996C>A (p.S399*)	Het	P	*
2	M	45	10	Pure	—	S	*SPAST*	c.1632C>G (p.Y544*)	Het	P	*
3	M	30	23	Pure	—	AD	*SPAST*	c.975_981del (p.A325fs)	Het	P	*
4	M	45	8	Pure	—	AD	*SPAST*	c.1795C>T (p.Q599*)	Het	P	*
5	M	1	49	Pure	—	AD	*SPAST*	c.1729-1G>C	Het	LP	Meijer et al. (2002)
6	F	39	8	Pure	—	S	*SPAST*	c.1664A>G (p.D555G)	Het	P	Shoukier et al. (2009)
7	F	22	14	Pure	—	S	*SPAST*	c.1005–2A>G	Het	P	McDermott et al. (2006)
8	F	46	8	Pure	—	S	*SPAST*	c.1176dupT (p.K393*)	Het	P	Svenstrup et al. (2009)
9	F	33	27	Pure	—	S	*SPAST*	c.683-?_870+? del (Exon 5 del)	Het	P	*
10	M	18	33	Complex	Peripheral neuropathy	AD	*SPAST*	c.1196C>T (p.S399L)	Het	P	Meijer et al. (2002), Dong et al. (2018)
11	M	39	9	Pure	—	S	*SPAST*	c.1750G>A (p.D584N)	Het	LP	Park et al. (2015)
12	M	27	6	Pure	—	AD	*SPAST*	c.1689delA (p.E563Dfs*2)	Het	LP	*
13	M	27	8	Pure	—	AD	*SPAST*	c.1217del (p.I460Kfs*6)	Het	LP	*
14	M	20	41	Pure	—	S	*SPAST*	c.1211T>C (p.F404S)	Het	VUS	*
15	F	1	57	Pure	—	AD	*ATL1*	c.1030C>A (p.P344T)	Het	LP	*
16	M	25	9	Pure	—	AD	*WASHC5*	c.1771T>C (p.S591P)	Het	VUS	Wang et al. (2014)
17	M	20	9	Complex	Cognition impairment	AR	*SPG11*	c.2990T>A (p.L997*); c.470T>C (p.L157P)	Het	P	*
									Het	VUS	*
18	F	25	34	Pure	—	AD	*REEP1*	c.146G>A (p.W49*)	Het	LP	Ma et al. (2020)
19	M	42	1	Complex	Peripheral neuropathy	AR	*B4GALNT1*	c.19G>A (p.A7T)	Hom	VUS	*

Abbreviation: AAO, age at onset; DD, disease duration; Het, heterozygote; Hom, homozygote; M, male; F, female; AD, autosomal dominant inheritance; S, sporadic; P, pathogenic; LP, likely pathogenic; VUS, variant uncertain significance; SPAST, spastin; ATL1, atlastin GTPase, 1; WASHC5, WASH Complex Subunit 5; SPG11, spatacsin; REEP1, Receptor Accessory Protein 1; B4GALNT1, Beta-1, 4-N-Acetyl-Galactosaminyltransferase 1. * Indicates an unreported variant.

The SPG4 causative gene *SPAST* was the most frequent gene in our cohort, accounting for 73.69% of genetically confirmed patients and 66.67% of AD-HSP patients (2 AD-HSP patients refused genetic testing). Some novel variants were first reported in our cohort, as (summarized in Figure 3C and Table 3) c.1211T > C is a missense variant and its clinical significance was considered unknown by ACMG rating. A variety of prediction software suggest that this mutation can cause destructive effects on protein function. Therefore, we considered it as a causative variant and further functional validation may be needed. Consistent with previous studies, subtle mutations were much more common in SPG4 in China (Dong et al., 2018).

Moreover, we identified an unreported variant in the SPG3A causative gene *ALT1*. SPG3A is the most common early-onset AD-HSP, presenting with slowly progressive p-HSP with an AAO usually less than 10 years, and is the second most frequently found in AD-HSP patients in China (Giordani et al., 2021; Kelly et al., 2022). The newly reported variant, c.1030C>A, is a missense mutation that may cause destructive effects on protein function according to prediction software. This variant has not been reported in HSP patients but other variant at the same amino acid position, p. P344S, is a known variant reported according to the HGMD (Fusco et al., 2012). The proband in our cohort (Case 15 in Figure 3D and Table 3) had a family history, and all of the variant carriers (patients) in his family had early AAO and presented with p-HSP.

We identified two unreported heterozygous mutations in *SPG11* in one patient (Case 16). SPG11 is the most common subtype of AR-HSP (Doleckova et al., 2022), and can present with c-HSP accompanied by cognitive deficits, dysarthria, intellectual disability, thin corpus callosum, and axonal peripheral neuropathy (Doleckova et al., 2022; Xing and Du, 2022). The patient had onset at the age of 22 years, mainly manifested as spasticity and weakness of the lower limbs, accompanied by mild cognitive impairment (Mini-Mental State Examination score: 24), which was basically consistent with the clinical manifestations of SPG11. The patient had a suspicious family history. His parents were not ill, but one brother of his mother had suspected similar symptoms and passed away with unknown cause. Pedigree verification was not performed due to a lack of samples. Although SPG11 is currently considered to be autosomal recessive, it has been previously reported that SPG11 patients carry compound heterozygous mutations (Chen et al., 2020; Meszarosova et al., 2020). Therefore, based on mutation pathogenicity prediction, the clinical manifestation of the patient and a literature review, we confirmed that compound heterozygous *SPG11* mutations were causative.

Other detected variants included a reported missense variant in *WASHC5* (SPG8), an unreported missense variant in *B4GALNT1* (SPG26), and a reported non-sense variant in *REEP1* (SPG31) (summarized in Table 3). The clinical significance of *B4GALNT1* c.19G>A (p.A7T) was considered uncertain by ACMG rating. The missense variant had a low sequency in the database, and prediction software suggests that this mutation can cause destructive effects on protein function. SPG26 is one of the causes of AR-HSP, and few cases have been reported worldwide (Wang et al., 2021). The AAO of our case was 42 years. He presented with c-HSP accompanied by peripheral neuropathy and anxiety, and his brother presented with lower limb spasticity accompanied by cognitive impairment but died at 27 years old with unknown cause. Although the AAO of the proband was not early onset but symptoms of the brothers were partly in line with complicated phenotypic features of SPG26 described in previous reports (Boukhris et al., 2013; Wang et al., 2021). Our case was found to carry a homozygous mutation and the variant was found to segregate with the disease in exiting family members (Case 19 in Figure 3D). Therefore, we considered it as a causative variant but further functional validation may be needed. Altogether, based on the results of mutation prediction software, cosegregation in the pedigree and a literature review, we concluded that these unreported variants might be pathogenic. More studies to clarify their pathogenicity and pathogenic mechanism are needed, such as functional validation experiments.

## Discussion

HSP is a group of inherited neurodegenerative diseases with high clinical and genetic heterogeneity. Clinical diagnosis and genetic diagnosis of the disease are both complex and difficult. We retrospectively collected and analyzed the clinical and genetic data of patients with a primary diagnosis and a final diagnosis of HSP based on a 14-year cohort from 2008 to 2022. With a deeper understanding of the disease and the development of technology, we can more easily diagnose and further study HSP. We summarized the clinical features and gene distribution in a large Chinese HSP cohort and reported 12 unreported suspected causative variants that may help complement the clinical and genetic map of HSP, especially in China.

Onset with lower limb symptoms, early AAO and family history are critical for the differential diagnosis of HSP and other uncommon nervous system disorders that may cause spastic paraplegia, and long-term periodic follow-up contributes to both confirming the diagnosis and understanding the genotypic spectrum of HSP. In this study, we enrolled 95 patients with a primary diagnosis of HSP, but only 54 patients were confirmed to have the diagnosis after long-term follow-up and genetic testing. The clinical diagnostic rate was 56.84%. The factor contributing to incorrect diagnosis is the high heterogeneity of HSP. On the one hand, there were large differences in patients from the AAO to clinical manifestations, including accompanying symptoms, in this cohort. Even in the same family, different patients can have different manifestations, consistent with the generally agreed clinical heterogeneity of HSP (Tesson et al., 2015). On the other hand, some neurodegenerative diseases have symptoms similar to HSP at the early stages, and the diversity of accompanying symptoms of HSP increases the difficulty in differential diagnosis (Regensburger et al., 2018). ALS was the most common differential diagnosis in our cohort (shown in Figure 2A), which is a relatively rapidly progressing fatal neurodegenerative disease, and patients often die in 3–5 years due to respiratory failure (Borghero et al., 2022), while HSP generally does not affect survival. The confirmed HSP patients had an earlier AAO and longer DD than the confirmed ALS patients in our cohort, consistent with previous studies and clinical practice (Cosottini et al., 2022). Primary lateral sclerosis (PLS) often presents with spastic paraplegia in elderly patients (predominantly male) and progresses to involve bulbar regions. However, up to 20% of PLS patients do not develop bulbar symptoms until 10 years after onset (Gordon et al., 2006). These diseases may sometimes be difficult to differentiate at the early stage, especially in patients with negative genetic results. Therefore, long-term periodic follow-up is needed.

Age of onset and diagnostic delay were associated with loss of independent walking. The early-onset patients (AAO<18 years) had a longer disease duration and longer diagnostic delay (time from onset to the initial visit). Similar to a previous study (Schule et al., 2016), we found that later AAO was associated with disability to walk unaided in the disease course. An earlier onset resulted in less severe disease. Our results suggested that timely visits are important especially for those with later onset. In our retrospective study, we quantified the disease severity by the four-stage functional mobility score instead of the Spastic Paraplegia Rating Scale (SPRS) which was more commonly used to assess the clinical severity of symptoms of HSP in some large prospective studies (Schule et al., 2016; Do et al., 2022). The Spastic Paraplegia Rating Scale was suggested to be a reliable and valid measure of disease severity (Schüle et al., 2006). However, it is not fully suitable for out-of-hospital follow-up of patients or retrospective studies because the assessment requires the presence of both patients and physicians. Our results suggested that the four-stage functional mobility score can be used for supplemental assessment of ambulatory function or as a group factor for statistical analysis, and SPRS may be used in our prospective study for further research.

The genotypic spectrum of HSP is still expanding and the associated pathogenic mechanism still needs to be explored. SPG4 was the most frequent genotype in our cohort and other cohorts, accounting for 43%–80% of AD-HSP (Dong et al., 2018; Varghaei et al., 2022). Although there is high heterogeneity of HSP in different regions, SPG4, caused by pathogenic mutations in *SPAST*, is still the most common genotype, likely accounting for the commonality of HSP with pathogenic mechanisms. We also reported two AR-HSP patients, one carrying compound heterozygous mutations in *SPG11*. Another AR-HSP patient was found to carry a homozygous mutation in *B4GALNT1* (SPG26). Few SPG26 patients have been reported either in China or other countries (Wang et al., 2021), and further studies are needed to elucidate the mechanism. Additionally, our study detected some rare genotypes of HSP in Chinese patients. SPG8 (*KIAA0196*) and SPG31 (*REEP1*) are rarely reported in China (Ma et al., 2018; Xing and Du, 2022)^,^ and here, we reported one case each.

Exploring and expanding the genetic spectrum of HSP remains a substantial challenge for clinicians and researchers. We did not analyze the association between genotype and clinical features, due to a lack of sample size. Although we found that loss of independent walking was not associated with whether the patient was genetically confirmed (Figure 3B), findings in a large cohort of 608 patients showed significant associations between genotypes and complicating symptoms and disease severity (Schule et al., 2016). Therefore, expanding the genetic spectrum will help in studying the natural history of HSP. The genetic diagnostic rate of our cohort was 35.18% (the proper pathogenicity of variants with uncertain clinical significance considered by ACMG rating was discussed above), and the portion of unsolved cases was particularly high in patients enrolled in earlier years in our cohort. NGS combined with MLPA greatly improved our ability to genetically diagnose HSP and met clinical needs. However, the diagnostic efficiency ranges from 29%–32% in diagnosis cohorts (D'Amore et al., 2018; Cui et al., 2020) and higher in research cohorts. One of the reasons may be the various mutation forms in causative genes. Researchers suggest using a two-step strategy using genetic panels followed by whole exome sequencing (WES), which would then increase the genetic diagnosis rate (Méreaux et al., 2022). Additionally, the genotypic spectrum of HSP is still expanding, and continuing to discover new HSP-related genes and developing new technologies are important.

The retrospective design of our study is a limitation and might have resulted in bias in the data collection. Second, some patients did not return to the hospital and had no detailed and professional physical, neuroimaging or neurophysiological re-examination in the follow-up period. Therefore, developing a follow-up strategy and seeking self-rated functional scales to quantify functional changes is needed in prospective studies in the future. Nevertheless, the study provides a better understanding of the clinical features and diagnostic process of HSP based on a relatively large and long-term cohort and expands the genotypic spectrum in Chinese HSP patients.

## Data Availability

The data presented in the study are deposited in the GenBank, accession number PRJNA934425. Further inquiries can be directed to the corresponding authors.
