# Supplementary material for: Clinical features and genetic spectrum of Chinese patients with hereditary spastic paraplegia: A 14-year study
Source: Front Genet. 2023 Feb 27;14:1085442. doi: 10.3389/fgene.2023.1085442 (PMC10008886; doi:10.3389/fgene.2023.1085442)
Supplement: Supplementary file 1 [file Table1.docx]

Supplemental Materials—Tables

**Table S1** Demographic and clinical features of 95 patients with primary diagnosis of HSP

| Clinical features | Number of cases, n (%) |
| --- | --- |
| Sex (Male, n) | 63 (66.32) |
| Family history | 28 (29.47) |
| Age at onset, y | 35.72±13.75 |
| Mean disease duration, y | 10.46±11.43 |
| Initial symptoms |  |
| Difficulty walking | 82 (86.32) |
| Difficulty walking combined with other symptoms | 8 (8.42) |
| Other symptoms | 5 (5.26) |
| Upper limb weakness/spasticity | 19 (20) |
| Lower limb weakness | 70 (73.69) |
| Sphincter problems | 13 (13.69) |
| Extrapyramidal symptoms | 9 (9.57)^b^ |
| Psychobehavioral problems/cognitive disorders | 4 (4.26) ^b^ |
| Signs | ^c^ |
| Upper limb hyperreflexia | 67 (72.04) |
| Lower limb hyperreflexia | 92 (98.92) |
| Upper limb weakness | 7 (7.53) |
| Lower limb weakness | 46 (49.46) |
| Dysarthria | 7 (7.53) |
| Ataxia | 15 (16.13) |
| Hypoalgesia | 4 (4.30) |
| Symmetry | 68 (71.58) |
| Peripheral neuropathy symptoms | 13 (14.29)^d^ |
| Electromyogram testing | 85 (89.47) |
| Combined with vertebral diseases |  |
| Cervical spondylosis | 18 (18.95) |
| Lumbar spondylosis | 8 (8.42) |
| Both | 1 (1.05) |
| Gene sequencing | 82 (86.32) |
| Combined with other nervous system diseases | 6 (6.32) |

a. Other neurological diseases: cerebrovascular disease, Hirayama disease, poliomyelitis, and pituitary tumors.

b. Data missing for 1 patient.

c. Data missing for 2 patients.

d. Data missing for 4 patients.

**Table S2** Detailed information of excluded cases (41 cases)

| **Case** | **Sex** | **AAO (years)** | **Age at initial visit (years)** | **Family history** | | **Initial affected limbs** | **Initial signs (hyperreflexia)** | | **EMG** | **Gene sequencing** | **Effective follow-up** | **Final diagnosis** |
| --- | --- | --- | --- | --- | --- | --- | --- | --- | --- | --- | --- | --- |
|  |  |  |  | |  |  | UL | LL |  |  |  |  |
| 1 | M | 25 | 26 | | × | LLL | √ | √ | 1 | × | √ | CDM |
| 2 | M | 55 | 57 | | × | BLL | NA | NA | NA | NA | × | Uncertain |
| 3 | M | 15 | 17 | | × | RLL | × | √ | 1 | NA | × | Uncertain |
| 4 | M | 42 | 45 | | × | BLL | √ | √ | 0 | × | √ | ALS |
| 5 | F | 44 | 51 | | √ | BLL | √ | √ | 1 | NA | √ | SCA |
| 6 | M | 47 | 47 | | × | BLL | √ | √ | 1 | √ | √ | ALS |
| 7 | M | 53 | 55 | | × | LLL | √ | √ | 0 | √ | √ | ALS |
| 8 | M | 26 | 46 | | × | LLL | × | √ | 0 | √ | √ | ALS |
| 9 | M | 37 | 38 | | × | RLL | √ | √ | 0 | √ | √ | ALS |
| 10 | M | 43 | 44 | | Uncertain | BLL | √ | √ | 1 | √ | √ | ALS |
| 11 | F | 51 | 52 | | √ | RLL | √ | √ | 1 | √ | √ | Uncertain |
| 12 | M | 24 | 28 | | × | RLL | × | √ | NA | √ | × | Uncertain |
| 13 | M | 50 | 55 | | × | LLL | √ | √ | 1 | √ | √ | Uncertain |
| 14 | F | 46 | 51 | | × | LLL | √ | √ | 0 | NA | × | Uncertain |
| 15 | F | 12 | 21 | | × | RLL | √ | √ | 0 | √ | × | Uncertain |
| 16 | M | 37 | 39 | | × | RLL | √ | √ | 1 | √ | √ | Uncertain |
| 17 | M | 30 | 31 | | × | RLL | √ | √ | 0 | √ | √ | PD |
| 18 | M | 41 | 44 | | × | RLL | √ | √ | 1 | √ | √ | Uncertain |
| 19 | F | 52 | 54 | | × | BLL | √ | √ | 0 | √ | √ | ALS |
| 20 | F | 38 | 39 | | × | LLL | √ | √ | 0 | √ | √ | ALS |
| 21 | F | 41 | 41 | | × | LLL | √ | √ | 0 | √ | √ | ALS |
| 22 | M | 41 | 42 | | × | RLL | √ | √ | 1 | √ | √ | ALS |
| 23 | M | 33 | 33 | | × | RLL | √ | √ | 1 | √ | × | Uncertain |
| 24 | F | 29 | 39 | | × | BLL | × | √ | NA | √ | × | Uncertain |
| 25 | M | 30 | 32 | | × | BLL | √ | √ | 0 | √ | × | Uncertain |
| 26 | F | 44 | 45 | | × | BLL | √ | √ | 0 | √ | √ | ALS |
| 27 | F | 57 | 59 | | × | LLL | √ | √ | 0 | √ | √ | PLS |
| 28 | M | 21 | 28 | | √ | BLL | √ | √ | NA | √ | √ | Restless leg syndrome |
| 29 | M | 54 | 62 | | × | BLL | √ | √ | 1 | √ | √ | Uncertain |
| 30 | M | 26 | 27 | | × | - | √ | √ | 1 | √ | √ | PD |
| 31 | F | 50 | 57 | | × | RLL | √ | √ | 0 | √ | √ | Peripheral neuropathy |
| 32 | F | 43 | 44 | | × | RLL | √ | √ | 0 | √ | √ | ALS |
| 33 | F | 42 | 43 | | × | RLL | × | √ | 1 | × | √ | Peripheral neuropathy |
| 34 | M | 36 | 36 | | × | RLL | √ | √ | 1 | √ | × | Uncertain |
| 35 | F | 47 | 50 | | × | RLL | √ | √ | 0 | √ | √ | PLS |
| 36 | M | 48 | 51 | | × | LLL | √ | √ | 1 | √ | √ | Peripheral neuropathy |
| 37 | F | 22 | 32 | | × | BLL | × | √ | 0 | √ | √ | SCA |
| 38 | F | 56 | 57 | | × | LLL | × | √ | 0 | √ | √ | ALS |
| 39 | M | 47 | 47 | | × | BLL | √ | √ | 0 | √ | √ | Chronic neurobrucellosis |
| 40 | F | 40 | 44 | | × | - | √ | √ | 0 | √ | √ | ALS |
| 41 | M | 23 | 24 | | × | BLL | √ | √ | 0 | √ | √ | Uncertain, HSP excluded |

Abbreviation：AAO=age at onset；DD= disease duration； M=male；F=female; BLL=bilateral lower limbs; LLL=left lower limb; RLL=right lower limb; UL=upper limb; LL=lower limb; NA= not available; ALS, amyotrophic lateral sclerosis; PLS, primary lateral sclerosis; PD, Parkinson’s disease; CDM, copper deficiency myelopathy; SCA, spinocerebellar ataxia. Effective follow-up indicates that sufficient evidence for final diagnosis has been collected or patients have not been lost to follow-up until now.
